# 1-(2-Phenyl­eth­yl)adamantane

**DOI:** 10.1107/S1600536810023251

**Published:** 2010-06-23

**Authors:** Michal Rouchal, Marek Nečas, Robert Vícha

**Affiliations:** a Department of Chemistry, Faculty of Technology, Tomas Bata University in Zlin, Nám. T. G. Masaryka 275, Zlín,762 72, Czech Republic; b Department of Chemistry, Faculty of Science, Masaryk University in Brno, Kamenice 5, Brno-Bohunice, 625 00, Czech Republic

## Abstract

In the title compound, C_18_H_24_, the adamantane cage consists of three fused cyclo­hexane rings in almost ideal chair conformations, with C—C—C angles in the range 108.0 (14)–111.1 (15)°. The phenyl and 1-adamantyl substituents adopt *anti* orientations with a C—C—C—C torsion angle of 177.10 (16)°. In the crystal packing, the mol­ecules are linked by weak C—H⋯π inter­actions into chains along the *a *axis.

## Related literature

The title compound was prepared according to a modified procedure of Adkins & Billica (1948 ▶). For some important properties of compounds bearing the adamantane scaffold, see: van der Schyf *et al.* (2009 ▶); van Bommel *et al.* (2001 ▶). For a related structure, see: Raine *et al.* (2002 ▶).
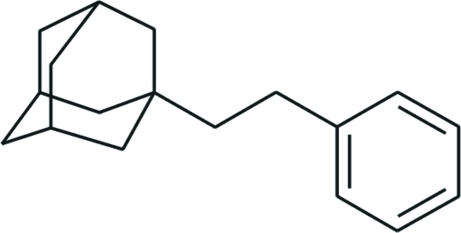


## Experimental

### 

#### Crystal data

C_18_H_24_
*M* *_r_* = 240.37
Orthorhombic, 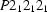

*a* = 6.4844 (5) Å
*b* = 7.5109 (5) Å
*c* = 28.5305 (19) Å
*V* = 1389.55 (17) Å^3^
*Z* = 4
Mo *K*α radiation
μ = 0.06 mm^−1^
*T* = 120 K
0.40 × 0.20 × 0.20 mm

#### Data collection

Kuma KM-4-CCD diffractometer
Absorption correction: multi-scan (*CrysAlis RED*; Oxford Diffraction, 2009 ▶) *T*_min_ = 0.924, *T*_max_ = 1.000
11994 measured reflections
1452 independent reflections
1277 reflections with *I* > 2σ(*I*)
*R*_int_ = 0.043

#### Refinement

*R*[*F* ^2^ > 2σ(*F* ^2^)] = 0.042
*wR*(*F* ^2^) = 0.127
*S* = 1.30
1452 reflections
163 parameters
H-atom parameters constrained
Δρ_max_ = 0.24 e Å^−3^
Δρ_min_ = −0.15 e Å^−3^

### 

Data collection: *CrysAlis CCD* (Oxford Diffraction, 2009 ▶); cell refinement: *CrysAlis RED* (Oxford Diffraction, 2009 ▶); data reduction: *CrysAlis RED*; program(s) used to solve structure: *SHELXS97* (Sheldrick, 2008 ▶); program(s) used to refine structure: *SHELXL97* (Sheldrick, 2008 ▶); molecular graphics: *ORTEP-3* (Farrugia, 1997 ▶) and *Mercury* (Macrae *et al.*, 2008 ▶); software used to prepare material for publication: *SHELXL97*.

## Supplementary Material

Crystal structure: contains datablocks global, I. DOI: 10.1107/S1600536810023251/ez2215sup1.cif

Structure factors: contains datablocks I. DOI: 10.1107/S1600536810023251/ez2215Isup2.hkl

Additional supplementary materials:  crystallographic information; 3D view; checkCIF report

## Figures and Tables

**Table 1:** Hydrogen-bond geometry (Å, °) *Cg*1 is the centroid of the C13–C18 ring.

*D*—H⋯*A*	*D*—H	H⋯*A*	*D*⋯*A*	*D*—H⋯*A*
C18—H18⋯*Cg*1^i^	0.95	2.64	3.529 (3)	156

Symmetry code: (i) 
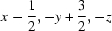
.

**Fig. 1.**
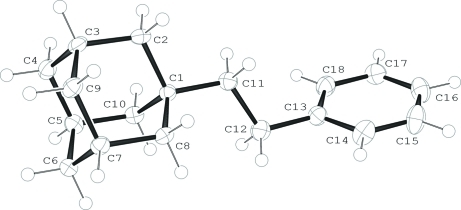
ORTEP diagram of the asymmetric unit showing the atom labelling scheme with atoms represented as 50% probability ellipsoids.

**Fig. 2.**
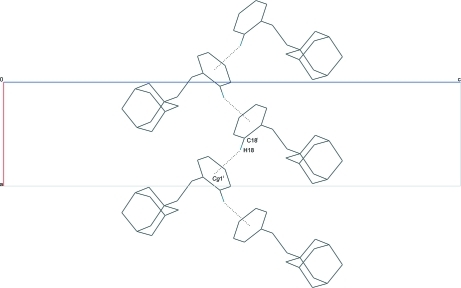
A partial view of the crystal packing viewed along the b-axis showing the arrangement of the molecules into chains parallel to the a-axis stabilized by weak C—H···π interactions (dotted lines). Cg1 is the center of gravity of C13–C18. H-atoms (except those which are involved in H-bonding) have been omitted for clarity. Symmetry codes: (i) -x + 1.5, -y + 1, z + 1/2; (ii) –x+2, y - 1/2, -z + 1/2.

## supplementary crystallographic information

### Comment

Adamantane is a molecule with an elegant structure and unique properties. The addition of the highly lipophilic adamantane cage to a known biologically active compound can significantly improve the pharmacokinetic profile of the resulting molecule, *e.g.* its oral bioavailability (van der Schyf *et al.* 2009). Moreover, the relatively stable host–guest interactions of the adamantane scaffold with β-cyclodextrin might increase the solubility of non-polar substances in polar media (van Bommel *et al.* 2001). Both these characteristics have an important role in drug design. This structure represents one of the few low-molecular-weight molecules bearing an adamantane moiety that has no polar function group. Therefore, this compound may be used as a standard molecule for investigations of non-polar interactions.

The asymmetric unit of the title compound consists of a single molecule (Fig. 1). The benzene ring is nearly planar with a maximum deviation from the best plane being 0.007 (2) Å for C16. The torsion angles describing mutual alignment of the 1-adamantyl and phenyl substituents C18—C13—C12—C11, C13—C12—C11—C1 and C12—C11—C1—C2 are -73.4 (2), -177.10 (16) and 179.59 (16)°, respectively. In the crystal packing, the molecules are arranged into chains parallel to the *a*-axis linked by weak C—H···π interactions (Fig. 2, Table 1).

### Experimental

The title compound was prepared according to a modified literature procedure published by Adkins & Billica (1948). 2-(1-Adamantyl)-2-benzyl-1,3-dithiane (0.33 mmol, 114 mg) was dissolved in 5 ml of dioxane and a large excess of Raney nickel catalyst was added to this solution. The reaction mixture was stirred and refluxed under Ar atmosphere. Further portions of Raney nickel were added until the starting material was completely consumed (monitored by GC). Subsequently, the Raney nickel was filtered off, the filtrate was diluted with water and extracted with diethyl ether. The combined organic layers were washed twice with brine and dried over Na_2_SO_4_. The required product was obtained after evaporation of solvent in vacuum as a colorless crystalline powder (72 mg, 91%, mp 318–324 K). The crystal used for data collection was grown by spontaneous evaporation from deuterochloroform at room temperature.

### Refinement

Hydrogen atoms were positioned geometrically and refined as riding using standard *SHELXTL* constraints, with their *U*_iso_ set to either 1.2*U*_eq_ of their parent atoms. In the absence of significant anomalous scattering, Friedel pairs were merged.

### Crystal data

**Table:**

C_18_H_24_	*D*_x_ = 1.149 Mg m^−^^3^
*M**_r_* = 240.37	Melting point: 321 K
Orthorhombic, *P*2_1_2_1_2_1_	Mo *K*α radiation, λ = 0.71073 Å
Hall symbol: P 2ac 2ab	Cell parameters from 5446 reflections
*a* = 6.4844 (5) Å	θ = 3.1–27.2°
*b* = 7.5109 (5) Å	µ = 0.06 mm^−^^1^
*c* = 28.5305 (19) Å	*T* = 120 K
*V* = 1389.55 (17) Å^3^	Block, colourless
*Z* = 4	0.40 × 0.20 × 0.20 mm
*F*(000) = 528	

### Data collection

**Table:**

Kuma KM-4-CCD diffractometer	1452 independent reflections
Radiation source: fine-focus sealed tube	1277 reflections with *I* > 2σ(*I*)
graphite	*R*_int_ = 0.043
Detector resolution: 0.06 mm pixels mm^-1^	θ_max_ = 25.0°, θ_min_ = 3.1°
ω scan	*h* = −5→7
Absorption correction: multi-scan (*CrysAlis RED*; Oxford Diffraction, 2009)	*k* = −8→8
*T*_min_ = 0.924, *T*_max_ = 1.000	*l* = −33→33
11994 measured reflections	

### Refinement

**Table:**

Refinement on *F*^2^	Primary atom site location: structure-invariant direct methods
Least-squares matrix: full	Secondary atom site location: difference Fourier map
*R*[*F*^2^ > 2σ(*F*^2^)] = 0.042	Hydrogen site location: inferred from neighbouring sites
*wR*(*F*^2^) = 0.127	H-atom parameters constrained
*S* = 1.30	*w* = 1/[σ^2^(*F*_o_^2^) + (0.0705*P*)^2^ + 0.0787*P*] where *P* = (*F*_o_^2^ + 2*F*_c_^2^)/3
1452 reflections	(Δ/σ)_max_ < 0.001
163 parameters	Δρ_max_ = 0.24 e Å^−^^3^
0 restraints	Δρ_min_ = −0.15 e Å^−^^3^

### Special details

**Table:**

Geometry. All e.s.d.'s (except the e.s.d. in the dihedral angle between two l.s. planes) are estimated using the full covariance matrix. The cell e.s.d.'s are taken into account individually in the estimation of e.s.d.'s in distances, angles and torsion angles; correlations between e.s.d.'s in cell parameters are only used when they are defined by crystal symmetry. An approximate (isotropic) treatment of cell e.s.d.'s is used for estimating e.s.d.'s involving l.s. planes.
Refinement. Refinement of *F*^2^ against ALL reflections. The weighted *R*-factor *wR* and goodness of fit *S* are based on *F*^2^, conventional *R*-factors *R* are based on *F*, with *F* set to zero for negative *F*^2^. The threshold expression of *F*^2^ > 2σ(*F*^2^) is used only for calculating *R*-factors(gt) *etc*. and is not relevant to the choice of reflections for refinement. *R*-factors based on *F*^2^ are statistically about twice as large as those based on *F*, and *R*- factors based on ALL data will be even larger.

### Fractional atomic coordinates and isotropic or equivalent isotropic displacement parameters (Å^2^)

**Table:**

	*x*	*y*	*z*	*U*_iso_*/*U*_eq_	
C1	0.8116 (4)	0.4428 (3)	0.14870 (8)	0.0172 (6)	
C2	0.6166 (4)	0.4772 (3)	0.17845 (9)	0.0207 (6)	
H2A	0.6402	0.5812	0.1991	0.025*	
H2B	0.4993	0.5052	0.1575	0.025*	
C3	0.5633 (4)	0.3148 (3)	0.20840 (8)	0.0206 (6)	
H3	0.4366	0.3400	0.2272	0.025*	
C4	0.5236 (4)	0.1543 (3)	0.17589 (9)	0.0232 (6)	
H4A	0.4887	0.0482	0.1949	0.028*	
H4B	0.4059	0.1803	0.1549	0.028*	
C5	0.7167 (4)	0.1176 (3)	0.14680 (8)	0.0212 (6)	
H5	0.6913	0.0137	0.1257	0.025*	
C6	0.8974 (4)	0.0749 (3)	0.17999 (9)	0.0222 (6)	
H6A	0.8640	−0.0316	0.1990	0.027*	
H6B	1.0230	0.0490	0.1615	0.027*	
C7	0.9363 (4)	0.2344 (3)	0.21230 (8)	0.0195 (6)	
H7	1.0545	0.2072	0.2337	0.023*	
C8	0.9892 (4)	0.3977 (3)	0.18208 (8)	0.0182 (6)	
H8A	1.0171	0.5010	0.2027	0.022*	
H8B	1.1154	0.3731	0.1637	0.022*	
C9	0.7437 (4)	0.2729 (4)	0.24146 (8)	0.0217 (6)	
H9A	0.7693	0.3756	0.2624	0.026*	
H9B	0.7097	0.1683	0.2611	0.026*	
C10	0.7703 (4)	0.2808 (3)	0.11744 (9)	0.0208 (6)	
H10A	0.6548	0.3076	0.0959	0.025*	
H10B	0.8941	0.2554	0.0983	0.025*	
C11	0.8577 (4)	0.6112 (3)	0.12008 (8)	0.0219 (6)	
H11A	0.7359	0.6377	0.1004	0.026*	
H11B	0.8750	0.7118	0.1421	0.026*	
C12	1.0478 (5)	0.6040 (4)	0.08826 (9)	0.0283 (7)	
H12A	1.1701	0.5727	0.1073	0.034*	
H12B	1.0284	0.5092	0.0646	0.034*	
C13	1.0866 (5)	0.7775 (4)	0.06375 (8)	0.0234 (6)	
C14	1.2499 (5)	0.8876 (4)	0.07581 (8)	0.0302 (7)	
H14	1.3389	0.8539	0.1007	0.036*	
C15	1.2855 (5)	1.0453 (4)	0.05228 (9)	0.0349 (8)	
H15	1.3969	1.1197	0.0615	0.042*	
C16	1.1596 (5)	1.0957 (4)	0.01530 (9)	0.0317 (7)	
H16	1.1860	1.2027	−0.0014	0.038*	
C17	0.9961 (5)	0.9891 (4)	0.00311 (9)	0.0285 (7)	
H17	0.9071	1.0238	−0.0217	0.034*	
C18	0.9604 (5)	0.8312 (4)	0.02684 (9)	0.0275 (7)	
H18	0.8477	0.7581	0.0178	0.033*	

### Atomic displacement parameters (Å^2^)

**Table:**

	*U*^11^	*U*^22^	*U*^33^	*U*^12^	*U*^13^	*U*^23^
C1	0.0173 (14)	0.0180 (13)	0.0163 (11)	−0.0001 (11)	0.0018 (10)	−0.0003 (10)
C2	0.0141 (13)	0.0236 (14)	0.0244 (13)	0.0037 (11)	0.0005 (12)	−0.0009 (11)
C3	0.0150 (14)	0.0257 (14)	0.0212 (12)	0.0006 (11)	0.0051 (11)	0.0004 (11)
C4	0.0187 (15)	0.0262 (14)	0.0247 (12)	−0.0028 (12)	−0.0013 (12)	0.0051 (11)
C5	0.0238 (15)	0.0188 (13)	0.0210 (12)	−0.0015 (12)	−0.0036 (11)	−0.0037 (11)
C6	0.0196 (14)	0.0202 (13)	0.0269 (13)	0.0012 (12)	0.0022 (12)	0.0028 (11)
C7	0.0157 (14)	0.0246 (14)	0.0184 (12)	0.0004 (12)	−0.0023 (11)	0.0037 (11)
C8	0.0155 (13)	0.0214 (13)	0.0177 (11)	−0.0004 (11)	0.0010 (11)	−0.0020 (11)
C9	0.0196 (15)	0.0278 (15)	0.0176 (11)	−0.0020 (13)	0.0016 (11)	0.0017 (10)
C10	0.0199 (15)	0.0237 (14)	0.0187 (11)	0.0010 (12)	−0.0007 (11)	−0.0012 (10)
C11	0.0216 (14)	0.0217 (14)	0.0223 (12)	0.0016 (12)	−0.0002 (12)	0.0007 (11)
C12	0.0275 (16)	0.0289 (15)	0.0284 (13)	0.0014 (14)	0.0050 (13)	0.0058 (12)
C13	0.0252 (16)	0.0248 (14)	0.0201 (12)	−0.0005 (12)	0.0029 (11)	0.0015 (11)
C14	0.0302 (16)	0.0415 (18)	0.0190 (12)	−0.0061 (15)	−0.0020 (12)	0.0030 (12)
C15	0.040 (2)	0.0352 (17)	0.0291 (14)	−0.0181 (15)	−0.0039 (14)	−0.0028 (13)
C16	0.0468 (19)	0.0250 (14)	0.0235 (13)	−0.0047 (15)	0.0047 (13)	0.0029 (12)
C17	0.0307 (17)	0.0317 (15)	0.0232 (13)	0.0030 (14)	−0.0002 (14)	0.0046 (11)
C18	0.0237 (16)	0.0283 (15)	0.0306 (14)	−0.0033 (13)	−0.0027 (12)	0.0000 (12)

### Geometric parameters (Å, °)

**Table:**

C1—C10	1.532 (3)	C8—H8B	0.9900
C1—C8	1.532 (3)	C9—H9A	0.9900
C1—C11	1.535 (3)	C9—H9B	0.9900
C1—C2	1.544 (3)	C10—H10A	0.9900
C2—C3	1.529 (3)	C10—H10B	0.9900
C2—H2A	0.9900	C11—C12	1.532 (4)
C2—H2B	0.9900	C11—H11A	0.9900
C3—C9	1.535 (4)	C11—H11B	0.9900
C3—C4	1.542 (3)	C12—C13	1.500 (4)
C3—H3	1.0000	C12—H12A	0.9900
C4—C5	1.527 (4)	C12—H12B	0.9900
C4—H4A	0.9900	C13—C14	1.387 (4)
C4—H4B	0.9900	C13—C18	1.393 (4)
C5—C10	1.525 (3)	C14—C15	1.381 (4)
C5—C6	1.540 (4)	C14—H14	0.9500
C5—H5	1.0000	C15—C16	1.387 (4)
C6—C7	1.533 (3)	C15—H15	0.9500
C6—H6A	0.9900	C16—C17	1.373 (4)
C6—H6B	0.9900	C16—H16	0.9500
C7—C9	1.528 (4)	C17—C18	1.385 (4)
C7—C8	1.538 (3)	C17—H17	0.9500
C7—H7	1.0000	C18—H18	0.9500
C8—H8A	0.9900		
			
C10—C1—C8	108.5 (2)	C1—C8—H8B	109.5
C10—C1—C11	112.24 (18)	C7—C8—H8B	109.5
C8—C1—C11	111.5 (2)	H8A—C8—H8B	108.0
C10—C1—C2	108.1 (2)	C7—C9—C3	109.08 (18)
C8—C1—C2	108.09 (18)	C7—C9—H9A	109.9
C11—C1—C2	108.3 (2)	C3—C9—H9A	109.9
C3—C2—C1	111.0 (2)	C7—C9—H9B	109.9
C3—C2—H2A	109.4	C3—C9—H9B	109.9
C1—C2—H2A	109.4	H9A—C9—H9B	108.3
C3—C2—H2B	109.4	C5—C10—C1	110.99 (19)
C1—C2—H2B	109.4	C5—C10—H10A	109.4
H2A—C2—H2B	108.0	C1—C10—H10A	109.4
C2—C3—C9	109.5 (2)	C5—C10—H10B	109.4
C2—C3—C4	108.97 (18)	C1—C10—H10B	109.4
C9—C3—C4	109.7 (2)	H10A—C10—H10B	108.0
C2—C3—H3	109.6	C12—C11—C1	116.3 (2)
C9—C3—H3	109.6	C12—C11—H11A	108.2
C4—C3—H3	109.6	C1—C11—H11A	108.2
C5—C4—C3	109.3 (2)	C12—C11—H11B	108.2
C5—C4—H4A	109.8	C1—C11—H11B	108.2
C3—C4—H4A	109.8	H11A—C11—H11B	107.4
C5—C4—H4B	109.8	C13—C12—C11	112.4 (2)
C3—C4—H4B	109.8	C13—C12—H12A	109.1
H4A—C4—H4B	108.3	C11—C12—H12A	109.1
C10—C5—C4	109.9 (2)	C13—C12—H12B	109.1
C10—C5—C6	109.4 (2)	C11—C12—H12B	109.1
C4—C5—C6	109.09 (18)	H12A—C12—H12B	107.9
C10—C5—H5	109.5	C14—C13—C18	117.6 (2)
C4—C5—H5	109.5	C14—C13—C12	122.0 (2)
C6—C5—H5	109.5	C18—C13—C12	120.4 (3)
C7—C6—C5	109.4 (2)	C15—C14—C13	121.2 (3)
C7—C6—H6A	109.8	C15—C14—H14	119.4
C5—C6—H6A	109.8	C13—C14—H14	119.4
C7—C6—H6B	109.8	C14—C15—C16	120.4 (3)
C5—C6—H6B	109.8	C14—C15—H15	119.8
H6A—C6—H6B	108.2	C16—C15—H15	119.8
C9—C7—C6	109.9 (2)	C17—C16—C15	119.2 (3)
C9—C7—C8	109.7 (2)	C17—C16—H16	120.4
C6—C7—C8	108.84 (18)	C15—C16—H16	120.4
C9—C7—H7	109.5	C16—C17—C18	120.3 (3)
C6—C7—H7	109.5	C16—C17—H17	119.9
C8—C7—H7	109.5	C18—C17—H17	119.9
C1—C8—C7	110.9 (2)	C17—C18—C13	121.3 (3)
C1—C8—H8A	109.5	C17—C18—H18	119.4
C7—C8—H8A	109.5	C13—C18—H18	119.4

### Hydrogen-bond geometry (Å, °)

**Table:**

*Cg*1 is the centroid of the C13–C18 ring.

**Table:**

*D*—H···*A*	*D*—H	H···*A*	*D*···*A*	*D*—H···*A*
C18—H18···Cg1^i^	0.95	2.64	3.529 (3)	156

Symmetry codes: (i) *x*−1/2, −*y*+3/2, −*z*.

## References

[bb1] Adkins, H. & Billica, H. R. (1948). *J. Am. Chem. Soc.* **70**, 695–698.

[bb2] Bommel, K. J. C. van, Metselaar, M. A., Werboom, W. & Reinhoudt, D. N. (2001). *J. Org. Chem.* **66**, 5405–5412. 10.1021/jo0102718 11485462

[bb3] Farrugia, L. J. (1997). *J. Appl. Cryst.* **30**, 565.

[bb4] Macrae, C. F., Bruno, I. J., Chisholm, J. A., Edgington, P. R., McCabe, P., Pidcock, E., Rodriguez-Monge, L., Taylor, R., van de Streek, J. & Wood, P. A. (2008). *J. Appl. Cryst.* **41**, 466–470.

[bb5] Oxford Diffraction (2009). *CrysAlis CCD* and *CrysAlis RED* Oxford Diffraction Ltd, Yarnton, England.

[bb6] Raine, A. L., Williams, C. M. & Bernhardt, P. V. (2002). *Acta Cryst.* E**58**, o1439–o1440.

[bb7] Schyf, C. J. van der & Geldenhuys, W. J. (2009). *Neurotherapeutics*, **6**, 175–186. 10.1016/j.nurt.2008.10.037 PMC5084265 19110208

[bb8] Sheldrick, G. M. (2008). *Acta Cryst.* A**64**, 112–122. 10.1107/S0108767307043930 18156677

